# Checkpoints in a Yeast Differentiation Pathway Coordinate Signaling during Hyperosmotic Stress

**DOI:** 10.1371/journal.pgen.1002437

**Published:** 2012-01-05

**Authors:** Michal J. Nagiec, Henrik G. Dohlman

**Affiliations:** 1 Department of Pharmacology, The University of North Carolina at Chapel Hill, Chapel Hill, North Carolina, United States of America; 2 Department of Biochemistry and Biophysics, The University of North Carolina at Chapel Hill, Chapel Hill, North Carolina, United States of America; Stanford University School of Medicine, United States of America

## Abstract

All eukaryotes have the ability to detect and respond to environmental and hormonal signals. In many cases these signals evoke cellular changes that are incompatible and must therefore be orchestrated by the responding cell. In the yeast *Saccharomyces cerevisiae*, hyperosmotic stress and mating pheromones initiate signaling cascades that each terminate with a MAP kinase, Hog1 and Fus3, respectively. Despite sharing components, these pathways are initiated by distinct inputs and produce distinct cellular behaviors. To understand how these responses are coordinated, we monitored the pheromone response during hyperosmotic conditions. We show that hyperosmotic stress limits pheromone signaling in at least three ways. First, stress delays the expression of pheromone-induced genes. Second, stress promotes the phosphorylation of a protein kinase, Rck2, and thereby inhibits pheromone-induced protein translation. Third, stress promotes the phosphorylation of a shared pathway component, Ste50, and thereby dampens pheromone-induced MAPK activation. Whereas all three mechanisms are dependent on an increase in osmolarity, only the phosphorylation events require Hog1. These findings reveal how an environmental stress signal is able to postpone responsiveness to a competing differentiation signal, by acting on multiple pathway components, in a coordinated manner.

## Author Summary

All cells can detect and respond to signals in their environment. The ability to interpret these signals with accuracy is needed for proper growth and differentiation. Moreover, cells must prioritize responses when confronted with competing signals. However the molecular mechanisms that govern signal prioritization are poorly understood. To address this question, we studied two signaling pathways in the genetic model organism budding yeast. Specifically we focused on the pheromone mating (differentiation) pathway and the high osmolarity glycerol (stress response) pathway. These pathways respond differently to each stimulus despite sharing pathway components. We find that cells must first adapt to stress before they can mate. At early times, the stress response cross-inhibits and dampens the pheromone response to suspend mating differentiation. Once cells adapt, the stress response ends and the differentiation program resumes. All signaling pathways that regulate cell fate decisions are interconnected to varying degrees. Our study highlights the importance of proper signal coordination in cell fate decisions, and it reveals new mechanisms that govern signal coordination within complex signaling networks.

## Introduction

Eukaryotic cells commonly employ mitogen activated protein kinases (MAPKs) to transduce extracellular signals and evoke intracellular responses [1]. MAPKs are a part of an evolutionarily-conserved three-tiered signaling cascade comprised of the MAPK, a MAPK kinase (MAPKK), and a MAPKK kinase (MAPKKK). In mammalian cells MAPKs respond to diverse stimuli including hormones, stresses, and cytokines. These different stimuli will in many cases activate a common MAPK. Conversely a single stimulus will often activate multiple MAPKs. Understanding how each stimulus and each response is coordinated is often obscured by the large number of components and the functional complexity of signaling networks [2].

**Figure 1:**
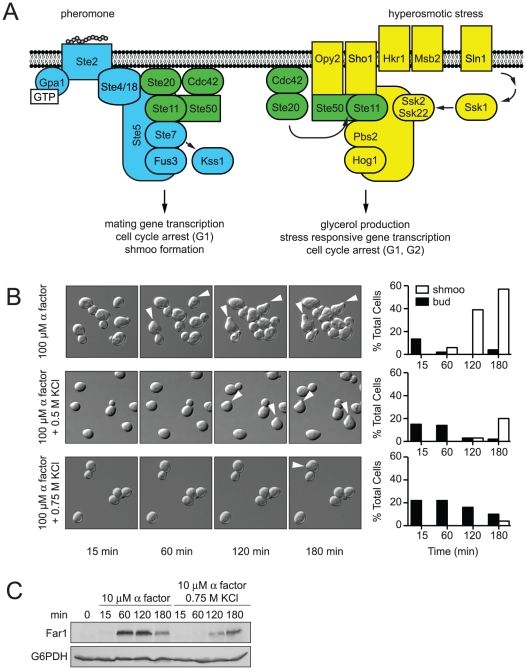
Hyperosmotic stress delays mating differentiation. (A) The mating pathway (blue) and the HOG pathway (yellow) share components (green). Overlapping lines indicate an interaction and activation of the downstream component, otherwise indicated by an arrow. The mating pathway [4], [57] is activated when mating pheromone binds the receptor (Ste2) and activates a G protein. The Gβγ subunit dimer (Ste4/18) recruits the MAPK complex comprised of the scaffold (Ste5), MAPK (Fus3), MAPKK (Ste7), MAPKKK (Ste11) and the Ste11 adaptor protein Ste50 to the plasma membrane [6], [7]. At the plasma membrane the small G-protein Cdc42 and the protein kinase Ste20 activate the assembled MAPK complex [58]. Activated Fus3 induces gene transcription, cell cycle arrest, and cytoskeletal rearrangement [8]–[10]. Mating pheromone also activates a second MAPK, Kss1. Kss1 primarily activates the haploid filamentous growth response, but also contributes to a full mating response [57]. The HOG pathway [59] is activated by hyperosmotic conditions. Two branches, *SHO1* and *SLN1*, detect hyperosmotic conditions. Hrk1 and Msb2 activate the *SHO1* branch [60], which shares components with the mating pathway [11]. The *SLN1* branch activates the protein kinase Ssk1 [61], which activates the partially redundant MAPKKKs Ssk2 and Ssk22 [62]. The *SHO1* and *SLN1* branches converge on the MAPKK, Pbs2, which serves as a scaffold for Ste11, Ssk2, Ssk22 and the MAPK, Hog1. Activated Hog1 induces glycerol production, gene transcription, and cell cycle arrest [12], [13], [33], [63], [64]. (B) Shmoo formation was visualized by microscopy after incubation of cells treated with 100 µM α factor, 100 µM α factor+0.5 M KCl, or 100 µM α factor+0.75 M KCl. The percentages of cells with shmoos or buds are shown. (C) Induction kinetics of Far1; wild-type cells were stimulated with 10 µM α factor and co-stimulated with 10 µM α factor+0.75 M KCl. Cell lysates were resolved by 7.5% SDS-PAGE and Far1-HA detected by immunoblotting with anti-HA antibodies. Glucose-6-phosphate dehydrogenase (G6PDH) served as a loading control.

MAPK pathways are also present in the unicellular eukaryote *Saccharomyces cerevisiae* (hereafter, yeast). As in higher eukaryotes, yeast use multiple MAPK pathways to respond to a variety of environmental signals [3]. The two best-characterized examples are the mating pathway and the high osmolarity glycerol (HOG) response pathway (detailed in Figure 1A) [4], [5]. The mating pathway operates through a cell-surface receptor that activates a canonical G protein heterotrimer. The activated G protein recruits Ste5, a scaffold protein that assembles and activates three component kinases: Ste11, Ste7 and the MAPK Fus3 [6], [7]. Active Fus3 promotes events leading to cell fusion including new gene transcription, cell cycle arrest and cytoskeletal rearrangements [8]–[10]. High osmotic stress activates Ste11 as well as Pbs2 and the MAPK Hog1 [11]. Active Hog1 promotes events leading to stress adaptation including increased glycerol production, cell cycle arrest and a pause in protein translation [12]–[17]. Individually, the two pathways have well-defined components, known points of regulation, and established measures of pathway output. Together, the pathways form a signaling network that is a model for the study of signal coordination.

The mating and HOG pathways share several components, yet exhibit remarkable signal fidelity when stimulated individually [3], [18] (Figure 1A, shared components highlighted in green). Hyperosmotic stress does not activate Fus3 or promote mating, and mating pheromones do not activate Hog1 or the HOG pathway. Such pathway fidelity may be maintained by two mechanisms: (i) pathway insulation and (ii) pathway cross-inhibition [19]. The pathway insulation model proposes that physical sequestration of components maintains specificity. For example, Ste11 exists in two scaffolded pools, one that selectively activates Fus3 and another that selectively activates Hog1 [20]. The pathway cross-inhibition model proposes that one pathway inhibits signaling by the competing pathway. For example, Hog1 is required to prevent the inadvertent activation of the mating response by hyperosmotic stress. When Hog1 is absent, or rendered catalytically inactive, hyperosmotic stress promotes mating. Thus it appears that Hog1 targets a component of the mating pathway to maintain fidelity [21]–[24]. However, previous studies were unsuccessful in identifying the substrate(s) of Hog1 in the mating pathway.

Thus the mechanisms that prevent cross-talk remain unresolved. A related and potentially more tractable question is how cells coordinate responses when the mating and HOG pathways are activated simultaneously. To address this question investigators have treated cells simultaneously with mating pheromone and hyperosmotic stress and used pathway-specific transcription reporters to monitor signaling in individual cells [24], [25]. One group reported that the responses to these inputs are mutually exclusive [25]. However a subsequent analysis identified a potential artifact, wherein cell death can produce a spurious signal in reporter assays that employ the red fluorescent protein [24]. In surviving cells reporters of both pathways are activated in proportion to their respective stimuli. Thus a single cell can respond to both hyperosmotic stress and pheromone, but how these responses are prioritized or coordinated remains to be determined.

In this study we establish that the hyperosmotic stress and mating pheromone signals are coordinated. Using a broad array of activity assays, conducted over various time scales, we show that Hog1 delays and dampens the response to pheromone and does so by two distinct mechanisms: (i) negative feedback phosphorylation of a shared component (Ste50) and (ii) feed-forward phosphorylation of a negative regulator of translation (Rck2). Thus, activated Hog1 invokes pathway cross-inhibition to delay the mating differentiation response. Mating differentiation resumes once cellular osmotic balance is restored and cross-inhibition is relieved. These studies provide a model of how a cell integrates competing signals to control cell fate.

## Results

### Hyperosmotic stress delays the mating response

A hallmark of the mating response is the appearance of a mating projection (shmoo formation), which functions as the eventual site of cell-cell fusion [26]. A hallmark of the osmotic stress response is a rapid but transient reduction in cell volume. This reduction occurs as water leaves the cell in order to equalize internal and external osmolarity. The cell then ramps up glycerol production to restore osmotic balance and cell volume [27]. These signaling pathways are likely to be coordinated as it was reported previously that a *decrease* in extracellular osmolarity disrupts efficient cell-cell fusion [28].

Here we investigated how an *increase* in extracellular osmolarity impinges on processes leading to fusion. Recent publications have examined the cell response following co-stimulation with pheromone and hyperosmotic stress, but these papers reached opposing conclusions [24], [25]. Moreover, the authors of the second paper conclude that the pathway insulation model is operative, but base their conclusion on the absence of evidence for the pathway cross-inhibition model. Both reports relied primarily on transcription-reporter assays conducted over a limited time scale. However, as detailed herein, hyperosmotic stress conditions can have confounding effects on transcription-reporter activity, particularly at early time points.

Both the mating and HOG pathways can be activated in a single cell [24]. Because both pathways can be activated simultaneously, it is evident that cross-inhibition does not operate between these pathways. However, several key questions remain unanswered. Most importantly, do cells mate normally under osmotic stress, or is the mating program delayed by the osmotic stress response? If so, how is the mating program delayed when both pathways can be activated simultaneously at the transcriptional level as shown previously [24]? Accordingly, we determined the effect of co-stimulation on multiple events and over a period of several hours; these events include MAPK activation, transcription induction, protein expression, cell differentiation and cell fusion.

We first examined if hyperosmotic stress interferes with mating. To this end we performed quantitative mating assays in the absence and presence of an osmolyte (0.5 M KCl). As shown in Table 1, hyperosmotic conditions decrease mating efficiency by about 64% after a 4-hour mating period. The observed decrease may be caused by events during stress adaptation that postpone mating. To allow stress adaptation we extended the mating period to 24-hours. In this case we observed a more modest 8% decrease in mating efficiency. These results suggest that cells are capable of efficient mating during hyperosmotic conditions, but only after a period of adaptation.

**Table 1:** Hyperosmotic stress decreases mating efficiency.

	Mating efficiency (%)*		
Mating duration (h)	YPD	YPD+0.5 M KCl	Effect of KCl#
4	77±14	28±5	0.36
24	83±16	76±13	0.92

Wild-type *MAT* **a** strains (BY4741) were mated with a wild-type *MAT*α strain (BY4742) on indicated media.

*Mating efficiency was calculated by dividing the number of diploid cells by the number of total cells after 4 h or 24 h mating.

# Mating efficiency on YPD+0.5 M KCl divided by the mating efficiency on YPD.

Mating requires that cells undergo G1 arrest and the formation of a mating projection. Thus we next investigated how hyperosmotic stress affects pheromone-induced shmoo formation. We stimulated *MAT* **a** cells with a saturating concentration of mating pheromone (α factor), or co-stimulated cells with pheromone and KCl. We then visualized and quantified the appearance of shmoos over time by microscopy (Figure 1B and Videos S1, S2, S3). As shown in Figure 1B, the addition of mating pheromone resulted in detectable shmoo formation by 60 minutes, with 60% of cells forming shmoos by 180 minutes. The simultaneous addition of osmolyte resulted in detectable shmoo formation only after 120 minutes, with just 20% of cells forming shmoos by 180 minutes. Addition of higher concentrations of osmolyte, 0.75 M (Figure 1B) or 1 M KCl (data not shown), further delayed shmoo formation. The duration of delay is likely a function of the time needed for cells to adapt, and could also account for the delay in mating noted above.

The data presented above reveal that salt stress delays shmoo formation and diminishes mating efficiency. We then considered whether there was a delay in other aspects of the pheromone response. To this end we monitored Far1. Far1 is induced by pheromone only during the G1 phase of the cell cycle, is required for cell polarization during mating, and is quickly degraded as cells exit G1 [29]–[32]. Thus, Far1 is a broad indicator of cellular events leading up to mating. Addition of mating pheromone alone resulted in detectable Far1 by 60 minutes, while co-stimulation with 0.75 M KCl delayed the appearance of Far1 to 120 minutes (Figure 1C). These findings indicate that the delay in shmoo formation corresponds with a delay in Far1 induction. Thus osmotic stress triggers a delay in the mating response, and this delay is evident at the molecular level as well as at the level of cellular morphogenesis and mating.

Co-stimulation of cells has previously been associated with cytotoxicity [24]. Under our experimental conditions however, nearly all cells survived co-stimulation and were able to resume cell division, as shown in Figure 1B, Videos S2 and S3. We also quantified cell viability using methylene blue staining. By this approach we observed cytotoxicity in ∼6% of the population after 2 hours of co-stimulation with KCl and pheromone (n = 2,632). These results indicate that the delay in Far1 induction, and the corresponding delay in morphogenesis and mating, is not the result of cell death. In contrast about one-third of cells co-stimulated with sorbitol and pheromone did not survive, as reported previously [24].

### Hyperosmotic stress dampens and delays transcriptional reporter activity

**Figure 2:**
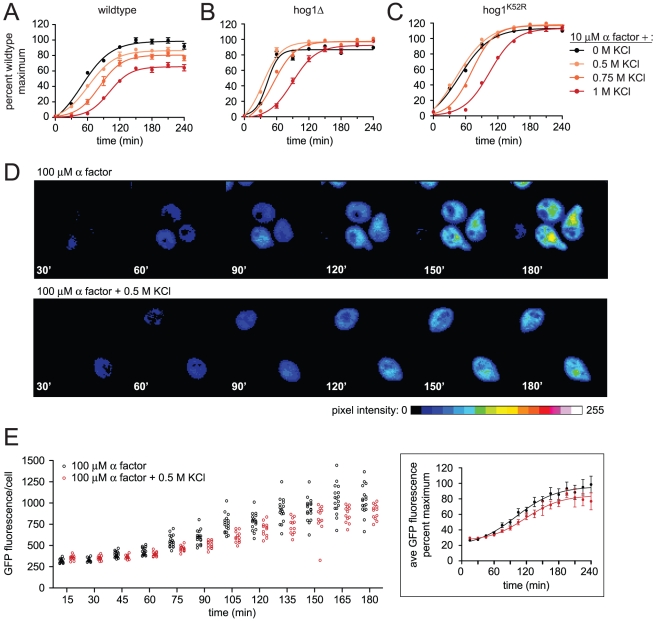
Hyperosmotic stress delays and dampens mating transcription. Transcriptional activation (β-galactosidase activity) was measured spectrofluorometrically every 30 min in (A) wild-type, (B) *hog1*Δ, and (C) *hog1^K52R^* cells transformed with a plasmid containing a pheromone-inducible reporter (*FUS1*-lacZ). Transcription was induced by the addition of 10 µM α factor, 10 µM α factor+0.5 M KCl, 10 µM α factor+0.75 M KCl, or 10 µM α factor+1 M KCl. Data are the mean ± SE of four individual colonies measured in quadruplicate and presented as percentage of wild-type maximum. Transcriptional activation (GFP expression) was measured by fluorescence microscopy in individual wild-type cells with an integrated pheromone-inducible reporter (*FUS1-GFP*). (D) Representative images of GFP expression in G1 cells stimulated by the addition of 10 µM α factor or 10 µM α factor+0.5 M KCl. Color spectrum indicates GFP pixel intensity as calculated using ImageJ. (E) Scatter plot of GFP fluorescence (average pixel intensity/cell area) in individual cells stimulated with 10 µM α factor or 10 µM α factor+0.5 M KCl. Insert is the average GFP intensity from the population of individual cells in (E), error bars indicate 95% CI.

Hyperosmotic stress activates Hog1 and induces genes required for adaptation [33]. During the immediate response to stress however, there is transient repression (<5 min) of overall gene transcription [34]. Moreover the duration of the delay correlates with the concentration of osmolyte and is prolonged in cells that lack Hog1 [34], [35]. Thus, transcription is regulated by Hog1-dependent and Hog1-independent mechanisms. We postulated that hyperosmotic stress might delay mating in part through a transient repression of transcription. Indeed, we have already shown that Far1 expression is delayed by salt stress; however Far1 abundance is also subject to stimulus-dependent ubiquitination and degradation [30]. To focus specifically on mating gene induction we used a reporter comprised of the β-galactosidase gene fused to the *FUS1* promoter (*FUS1*-lacZ). The *FUS1* gene is among the most strongly induced genes during the mating response [9]. As shown in Figure 2, cells stimulated with mating pheromone reached half maximum (t_½ max_) β-galactosidase activity at roughly 50 minutes (Figure 2A and Table S1). The addition of 0.75 M KCl increased the t_½ max_ by ∼67% and dampened the maximum response by ∼17%. The effects of salt were dose-dependent; with increasing concentrations the delay and dampening became progressively more pronounced.

To distinguish Hog1-dependent and Hog1-independent effects on the transcription response, we measured *FUS1* induction in cells lacking *HOG1* (*hog1*Δ) as well as in cells expressing a catalytically deficient mutant, Hog1^K52R^. These cells were then stimulated with pheromone, or co-stimulated with pheromone and KCl. Similar to wild-type cells, co-stimulation of *hog1*Δ cells increased the t_½ max_ for *FUS1* induction (Figure 2B and Table S2). However, unlike wild-type cells, co-stimulation of *hog1*Δ cells did not dampen the maximum response. Thus Hog1 contributes to the reduction in transcription response. Also, the change in t_½ max_ was less pronounced in *hog1*Δ cells compared to wild-type cells, suggesting that Hog1 is at least partly responsible for the delay in mating transcription (Table S2). Hog1^K52R^ cells showed an intermediate t_½ max_ and increased maximum response (Figure 2C and Table S3). This result was anticipated given that Hog1^K52R^ cells exhibit an intermediate level of cross-inhibition and sensitivity to osmotic stress [22], [23], [36]. The non-ionic osmolyte sorbitol was 5-fold more likely to cause cell death but otherwise acted much like KCl (Figure S1 and Table S1). Taken together these results support the view that osmotic stress attenuates mating transcription, and does so by Hog1-dependent and Hog1-independent mechanisms. Moreover, no additional effects associated with hyperosmotic stress were observed on the mating response (Text S1, Figure S2, Table S4, and Figure S3).

The transcriptional reporter *FUS1*-lacZ measures average differences in a population of cells. However this approach could mask larger differences within a subpopulation of cells, such as those in G1 phase where mating occurs [37]. To determine whether salt diminishes the mating response in a cell cycle dependent manner, we monitored transcription activity in single cells using a green fluorescent protein-based reporter (*FUS1*-GFP). To avoid any confounding effects of cell cycle-arresting agents, we specifically examined unbudded (G1 phase) cells in an otherwise asynchronous population. As shown in Figure 2D salt delays both GFP production and shmoo formation. Measurements of average pixel intensity among single-cells showed that the distribution of responding cells was uniform whether stimulated with pheromone or co-stimulated with pheromone and KCl (Figure 2E). Moreover, the salt mediated delay observed for cells in G1 phase (∼18 min) was similar to that of an asynchronous cell population (∼12 min) (Figure 2A, 2E and Tables S1, S5). Taken together, single-cell measurements corroborate the observations made using the population-based reporter for mating pathway output.

### Hyperosmotic stress dampens mating MAPK activation

**Figure 3:**
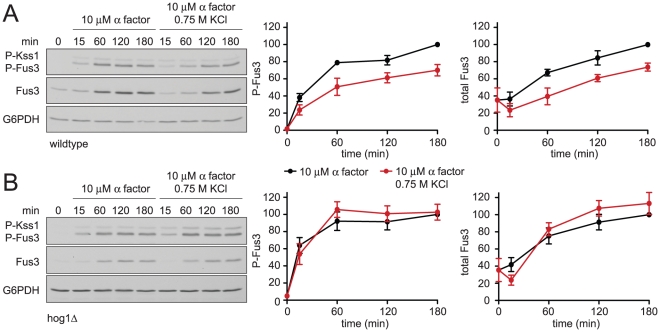
Hyperosmotic stress dampens mating MAPK activation and induction. (A) Activation and induction kinetics of Fus3; wild-type cells were stimulated with 10 µM α factor or co-stimulated with 10 µM α factor+0.75 M KCl. Cell lysates were resolved by 12.5% SDS-PAGE. Phospho-Fus3 (P-Fus3) and phospho-Kss1 (P-Kss1) were detected by immunoblotting with phospho-p44/p42 antibodies, which recognize the dually phosphorylated and activated form of Fus3 and Kss1. Total Fus3 abundance was determined with Fus3 antibodies. G6PDH served as a loading control. All primary antibodies were recognized by chemiluminescent detection and quantified by scanning densitometry (ImageJ). The panels to the right show averaged scanning densitometry of three individual experiments. Error bars represent ± SEM. Co-stimulation dampened P-Fus3 by 29.9%±6.6% and total Fus3 by 26.2%±4.7% at 180 min. (B) *hog1*Δ cells treated as in A.

Mating pheromones activate Fus3 and induce the transcription of genes required for haploid cell fusion. We have observed that Hog1 dampens and delays the mating transcription response. To determine how Hog1 limits the activation of Fus3 we monitored its activity directly, by immunoblotting with an antibody that recognizes the dually-phosphorylated, fully-active form of the kinase (phospho-Fus3) [38]. As shown in Figure 3, co-stimulation with KCl reduced phospho-Fus3 by one-third compared to cells treated with pheromone alone. Pheromone also induces the expression of the *FUS3* gene [9], [39]. To determine the effect of KCl on Fus3 production we quantified Fus3 protein levels with a Fus3-specific antibody. As with phospho-Fus3, total Fus3 was reduced by one third in co-stimulated cells. Thus hyperosmotic stress leads to dampened induction of Fus3 and a concomitant reduction in phospho-Fus3. We then conducted the same experiment in cells lacking Hog1 (Figure 3B). In this case, we found no effect of salt co-stimulation on phospho-Fus3 or Fus3. These data indicate that Hog1 regulates mating by dampening Fus3 production and, consequently, Fus3 activity. Thus Hog1 has a role in limiting gene induction and mating, and may do so by targeting a component downstream of Fus3.

**Figure 4:**
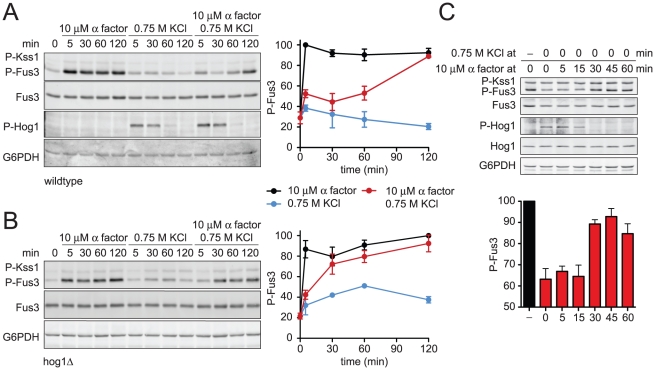
Hyperosmotic stress dampens Fus3 activation in a Hog1-dependent manner. (A) Activation kinetics of Fus3 and Hog1; wild-type cells transformed with plasmid-borne *GAL1-FUS3* were grown in SC and 2% galactose followed by stimulation with 10 µM α factor, 0.75 M KCl, or co-stimulation with 10 µM α factor+0.75 M KCl. Cell lysates were resolved by 12.5% SDS-PAGE. P-Fus3 and P-Kss1 were detected with phospho-p44/p42 antibodies. P-Hog1 was detected with phospho-p38 antibodies. Total Fus3 and Hog1 were detected with Fus3 and Hog1 antibodies. G6PDH served as a loading control. All primary antibodies were recognized by fluorescently labeled secondary antibody, detected by fluorescence scanner (Typhoon Trio) and quantified by scanning densitometry (ImageJ). The panel to the right shows averaged scanning densitometry of four individual experiments. Error bars represent ± SEM. Co-stimulation dampened P-Fus3 by 47.6%±2.2% at 5 min and 47.5%±6.6% at 30 min. (B) *hog1*Δ cells transformed with *GAL1-FUS3* treated as in A. Co-stimulation dampened P-Fus3 by 44.3%±7.4% at 5 min and 7.4%±10.9% at 30 min. (C) Sequential stimulation of Hog1 and Fus3. Wild-type cells grown in SC with 2% dextrose stimulated with 0.75 M KCl, and after an indicated period of stress adaptation stimulated with 10 µM α factor for an additional 15 min. Error bars represent ± SEM.

Fus3 is part of a positive feedback loop: the activation of Fus3 by mating pheromone leads to induction of more Fus3, which is subsequently activated by pheromone. We have shown above that Hog1 acts downstream of Fus3, by limiting induction of the protein. We then considered whether Hog1 acts upstream of Fus3, by limiting activation of the kinase. To exclude effects of hyperosmotic stress on Fus3 induction we replaced the native (pheromone-inducible) promoter with the galactose-regulated *GAL1* promoter. As expected, we found that cells grown in galactose stably express Fus3, with no induction in the presence of pheromone. However, co-stimulation with salt reduced phospho-Fus3 to nearly one-half of that in cells treated with pheromone alone, even as Fus3 abundance remained unchanged (Figure 4A). In contrast, co-stimulation did not alter phospho-Fus3 in the absence of Hog1 (Figure 4B), except for a small reduction at the earliest (5 min) time point. The effect of salt at 5 minutes was reported by others to be independent of Hog1. Specifically, cells lacking Hog1 require more time to reach ionic equilibrium [34]. Therefore the reduction seen at 5 minutes in *hog1*Δ cells is likely associated with the extended time required for adaptation to the mechanical and ionic stress caused by cell shrinking. Thus hyperosmotic stress dampens Fus3 activation. The reduction at early time points is evident with or without Hog1, while the reduction at later time points (30 and 60 min) is Hog1-dependent (Figure 2 and Figure 4).

Hog1 is activated rapidly following salt stress and then becomes inactive once the cells have adapted [14]. Fus3 is activated by pheromone, but activation in this case is delayed as long as Hog1 is active. Thus it appears that Fus3 cannot fully respond to pheromone until the cells have adapted to osmotic stress conditions. To investigate this behavior further we treated cells with KCl for various times, followed by treatment with pheromone for 15 minutes. Once again we observed that Fus3 activity is restricted as long as Hog1 is active (Figure 4C). Taken together, our findings indicate that Hog1 regulates mating at two points in the pathway, one downstream of Fus3 that limits protein induction and another upstream of Fus3 that limits kinase activation.

### Constitutively active Hog1 dampens mating MAPK activation

**Figure 5:**
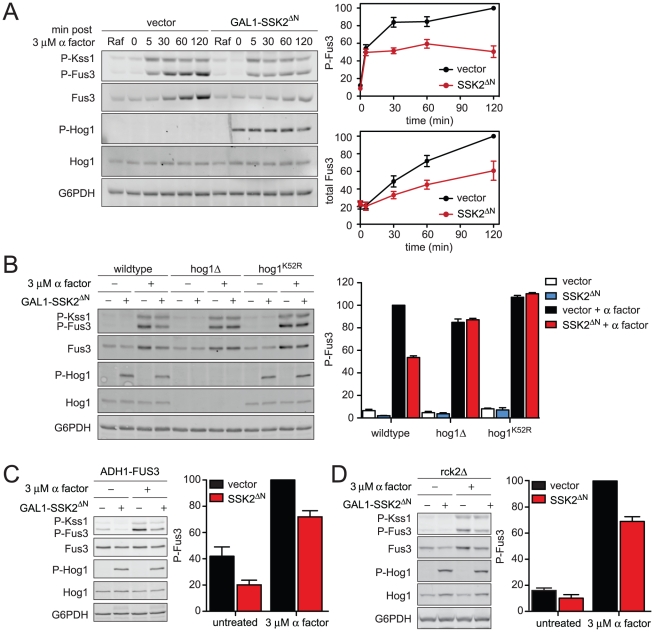
Constitutively active Hog1 dampens Fus3 activation and induction. (A) Activation kinetics of Fus3 with constitutively active Hog1; wild-type cells transformed with vector control or plasmid-borne *GAL1*-*SSK2^ΔN^* were grown in SC media with 2% raffinose (Raf). Ssk2^ΔN^ expression was induced by addition of 2% galactose for 60 min followed by addition of 3 µM α factor for 30 min. Cell lysates were resolved by 12.5% SDS-PAGE. P-Fus3 and P-Kss1 were detected with phospho-p44/p42 antibodies. P-Hog1 was detected with phospho-p38 antibodies. Total Fus3 and Hog1 were detected with Fus3 and Hog1 antibodies. G6PDH served as a loading control. All primary antibodies were recognized by fluorescently labeled secondary antibody, detected by fluorescence scanner (Typhoon Trio) and quantified by scanning densitometry (ImageJ). The panels to the right show averaged scanning densitometry of four individual experiments. Error bars represent ± SEM. P-Hog1 reduced P-Fus3 by 49.4%±6.7% at 120 min. (B) Wild-type, *hog1*Δ, and, *hog1^K52R^* cells transformed with *GAL1*-*SSK2^ΔN^* or parent vector control were grown in SC and 2% galactose for 60 min followed by addition of 3 µM α factor or left untreated for 30 min. (C) *fus3*Δ cells transformed with *ADH1*-*FUS3* and *GAL1*-*SSK2^ΔN^* or vector were grown and stimulated as in B. P-Hog1 (*SSK2^ΔN^*) reduced P-Fus3 by 30.7%±3.2%. (D) *rck2*Δ cells transformed with *GAL1*-*SSK2^ΔN^* or vector were grown and stimulated as in B. P-Hog1 reduced P-Fus3 by 31.0%±6.2%.

Cells utilize Hog1-dependent and Hog1-independent mechanisms to adapt to hyperosmotic stress. To focus exclusively on Hog1-dependent mechanisms we activated the kinase directly, without an osmolyte. To this end we introduced a constitutively active MAPKKK, Ssk2^ΔN^ [40], [41]. Ssk2 is a component of the SLN1-branch of the HOG pathway and is not shared with the mating pathway. Thus expression of Ssk2^ΔN^ activates Hog1 but does not affect Fus3 directly. First, we measured the effect of constitutively-activated Hog1 on pheromone-activated Fus3 over time (Figure 5A). Under these conditions Fus3 activation was reduced by up to 50%, comparable to the reduction observed with KCl (Figure 3A). Hog1 also limited expression of total Fus3 protein. As an additional control we tested mutants lacking Hog1 expression or Hog1 catalytic activity. In this case we observed no change in phospho-Fus3 or Fus3 abundance (Figure 5B). Thus Fus3 can be regulated by Hog1 even in the absence of hyperosmotic stress. Together these results reveal that Hog1 activation is necessary and sufficient to dampen Fus3 activation.

As noted above, induction of Fus3 can confound any analysis of Fus3 activation. To distinguish the effects of Hog1 on Fus3 induction and Fus3 phosphorylation, again we replaced the native *FUS3* promoter. In this case we used the strong constitutive promoter from *ADH1* (*ADH1*-*FUS3*) instead of the *GAL1* promoter used above, so as to prevent promoter competition and to ensure consistent levels of Hog1 activation. Under these conditions, constitutively active Hog1 reduced phospho-Fus3 by about one-third, somewhat less than the one-half reduction obtained in cells with the native *FUS3* promoter (Figure 5C). We obtained similar results using *GAL1-FUS3* (Figure S4) instead of *ADH1-FUS3* (Figure 5C). Thus Hog1 dampens Fus3 activation, even when expression is permanently elevated. When Fus3 is expressed from the native promoter, activation is dampened even further. These findings confirm that Fus3 induction and Fus3 phosphorylation are diminished by at least two distinct mechanisms that require Hog1. Each of these mechanisms is considered below.

### Rck2 is phosphorylated by Hog1 to limit mating signal during co-stimulation

To establish the mechanisms of pathway cross-inhibition we began with the target of Hog1 that limits Fus3 production. The induction of Fus3 requires transcription of the *FUS3* gene and translation of the corresponding mRNA. Hog1 phosphorylates and activates the protein kinase, Rck2 [42]. Activated Rck2 phosphorylates the yeast elongation factor, EF2, and thereby transiently represses translation [43], [44]. Thus we considered whether Rck2 regulates the production of Fus3 under osmotic stress conditions. To test the hypothesis, we constitutively activated Hog1 in the absence of *RCK2*, and in the presence or absence of pheromone (Figure 5D). Under these conditions, constitutively active Hog1 reduced phospho-Fus3 by about one-third, somewhat less than the one-half reduction obtained in cells that express Rck2 (compare Figure 5B and Figure 5D). Thus Rck2 is partially responsible for the diminished Fus3 response. Taken together these results suggest that Rck2 generally represses translation in response to Hog1, which results in diminished production of Fus3. More broadly these results provide evidence that the consequent repression of Fus3 translation contributes to decreased pheromone responsiveness following hyperosmotic stress.

### Ste50 is phosphorylated by Hog1 to limit mating signal during co-stimulation

**Figure 6:**
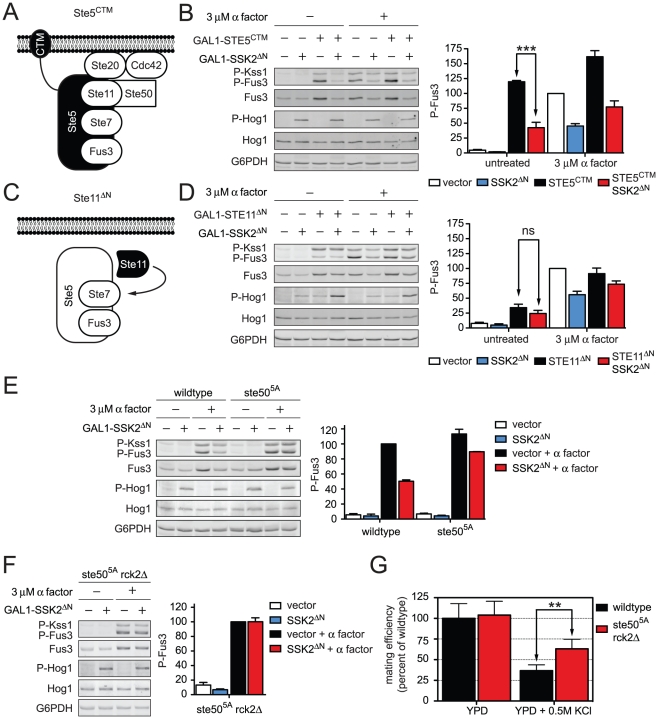
Hog1 dampens Fus3 activation by targeting Ste50. Constitutive activators of mating pathway highlighted in black: (A) Ste5^CTM^, a C-terminal transmembrane domain (CTM) tethers Ste5 to the plasma membrane allowing MAPK activation without receptor or G-protein. (B) Wild-type cells transformed with *GAL1*-*STE5^CTM^*, *GAL1*-*SSK2^ΔN^* or parent vector controls were grown in 2% galactose for 60 min followed by addition of 3 µM α factor or left untreated for 30 min. Cell lysates were resolved by 12.5% SDS-PAGE. Statistical significance was calculated using two-way ANOVA. ***, p<0.001. (C) Ste11^ΔN^, constitutively active amino-terminus truncation mutant of Ste11, allowing activation without binding the upstream activator Ste20, scaffold Ste5, or adaptor Ste50. (D) Wild-type cells transformed with *GAL1*-*STE11^ΔN^*, *GAL1*-*SSK2^ΔN^* or vector were grown in 2% galactose for 2.5 h followed by addition of 3 µM α factor or left untreated for 30 min. Statistical significance was calculated using two-way ANOVA, ns – not significant, p>0.05. (E) Wild-type and *ste50^5A^* cells grown and treated as in B. (F) *ste50^5A^ rck2*Δ cells grown and treated as in B. (G) Quantitative mating assay, indicated strains were mated with wild-type *MAT*α strain for 4 h on YPD or YPD+0.5 M KCl. Statistical significance was calculated using two-way ANOVA. **, p<0.01.

The data presented above indicate that Hog1 limits Fus3 activity in two ways. First, Hog1 phosphorylates Rck2 and suspends translation of mating pathway components. We have also presented evidence that Hog1 inhibits an upstream activator of the mating MAPK. To identify the second target of Hog1 we employed a genetic epistasis approach. First we determined if constitutively active Hog1 dampens the mating pathway at the level of the three-tiered MAPK cascade. Under normal circumstances the mating signal is initiated by the recruitment of the MAPK scaffold Ste5 to the plasma membrane [45]. Ste5 can be tethered permanently to the plasma membrane via fusion to a carboxy-terminal transmembrane domain (CTM), thus bypassing the need for pheromone, receptor, and G protein in pathway activation (Figure 6A) [7]. In cells that co-express *GAL1*-*STE5^CTM^* and *GAL1*-*SSK2^ΔN^*, phospho-Fus3 was dampened, similar to that seen with pheromone and *GAL1*-*SSK2^ΔN^* (Figure 6B). These data indicate that Hog1 acts on a component downstream of the G protein. We likewise observed dampening of phospho-Kss1, which is also activated by pheromone. These data suggest that the putative Hog1 target is upstream of both Fus3 and Kss1. Taken together these results narrowed the likely target to a handful of components associated with Ste5: the MAPKKK Ste11, its adaptor protein Ste50, its activators Cdc42 and Ste20, and its substrate Ste7 (Figure 1A).

Ste50 is required for full activation of Hog1, Fus3, and Kss1. We and others have demonstrated that Hog1 phosphorylates Ste50 during hyperosmotic conditions. Moreover, the phosphorylation of Ste50 leads to functional downregulation of Hog1 [46], [47]. Given this precedent, we hypothesized that phosphorylation of Ste50 leads to the downregulation of Fus3 and Kss1. To test the role of Ste50, we activated the mating pathway using a truncated form of Ste11; Ste11^ΔN^ lacks the kinase auto-inhibitory domain [48], and also lacks the Ste50 binding domain (Figure 6C) [49]. Thus Ste11^ΔN^ is both constitutively active and refractory to Ste50. As shown in Figure S5, Ste50 is not required for pathway activation by Ste11^ΔN^ even while it is required for full activation by Ste5^CTM^. We had postulated that Fus3 activity is dampened when Ste50 is phosphorylated. Accordingly, Fus3 should not be affected by Hog1 or Ste50 when the pathway is activated through Ste11^ΔN^ (Figure 6D). Under these conditions, Fus3 is fully activated, consistent with our prediction. Presumably Ste7 is also activated under these conditions, although currently we are not able to monitor its activity directly. Taken together these data suggest that Hog1 limits the mating signal at the level of Ste50. The mating and hyperosmotic stress signals are integrated by Ste50, which in turn regulates the shared MAPKKK, Ste11.

Next we sought to establish whether phosphorylation of Ste50 by Hog1 was responsible for pathway cross-inhibition. To this end we used a mutant of Ste50 (Ste50^5A^) where five MAPK sites have been changed to alanine, thereby abrogating phosphorylation by Hog1 [46], [47]. Consistent with our prediction Ste50^5A^ restored the ability of pheromone to activate Fus3, even under conditions of constitutive Hog1 activation (Figure 6E). Fus3 was not fully activated however, presumably because Hog1 could still target Rck2. When we deleted *RCK2* from the *ste50^5A^* strain we were able to attain full activation of Fus3 (Figure 6F and Figure S6). Thus Hog1 limits mating through the phosphorylation of at least two proteins, Ste50 and Rck2. More generally, these results reveal that cross-inhibition occurs through a combination of feedback and feedforward phosphorylation events.

Finally we aimed to determine the biological significance of Fus3 cross-inhibition by Hog1. As shown in Table 1, mating efficiency of wild-type cells is reduced by hyperosmotic conditions, presumably by Hog1-dependent and Hog1-independent mechanisms. To determine the contribution of Hog1 we performed quantitative mating assays in the presence or absence of the two cross-inhibition targets, either alone or in combination. Whereas mating efficiency is diminished in the presence of salt, mating was partially restored in the *ste50^5A^ rck2*Δ mutant strain (Figure 6G and Table S6). The partial rescue suggests that other mechanisms may be operative, or perhaps mating fails because the cells are still responding to stress. Fus3 activation was likewise restored in these mutant cells (Figure S7). Together, our results show that Hog1 inhibits Fus3 induction and activation, and these processes serve to delay mating until the cells have fully adapted to osmotic stress conditions.

## Discussion

All cells have the ability to detect changes in their environment and to produce responses appropriate to that stimulus. Our focus here was on two signals that may produce incompatible responses; one that triggers mating differentiation and a second that promotes adaptation to hyperosmotic stress. More specifically, we investigated the ability of yeast cells to coordinate responses to mating pheromones and high salt. We found that in co-stimulated cells, adaptation to hyperosmotic stress takes precedence.

### Stress adaptation suspends mating

Prior to our investigations, it was established that Hog1 activation is proportional to the severity of the hyperosmotic stress [50]. Furthermore, the duration of Hog1 activation is tightly correlated with glycerol production and a return to osmotic equilibrium [14]. Thus, hyperosmotic stress and cell adaptation dictate the level and duration of Hog1 activity. Our results support a model where Hog1 suspends the mating response until cells are fully adapted. In particular, we found that Hog1 dampens and delays Fus3 activation, and that the duration of delay is proportional to the severity of the hyperosmotic stress. Just as transient activation of Hog1 leads to transient inhibition of Fus3, persistent activation of Hog1 leads to persistent inhibition of Fus3.

### Hog1 regulates Fus3 activation and induction

It was established previously that osmotic stress results in a general *inhibition* of gene transcription. In cells that lack Hog1, transcription initiation is delayed further [34]. These results show the broad negative effects of salt on gene transcription and point to Hog1 as the primary mediator of the stress response. Paradoxically, cells that lack Hog1 exhibit a stress-mediated *increase* in the transcription of mating genes. These findings point to a special function for Hog1 in limiting the mating pathway. A major challenge has been to understand how Hog1 regulates Fus3, in addition to any Hog1-independent processes that might affect Fus3 induction. This was achieved by (i) constitutive expression of Fus3 (via promoter replacement) and (ii) direct activation of Hog1 (via Ssk2^ΔN^). Ultimately these approaches allowed us to identify Ste50 and Rck2 as important targets of Hog1. Phosphorylation of these proteins accounts for delayed mating responses during co-stimulation. However, other targets of Hog1 are likely. In the absence of Hog1, high osmolarity activates the transcriptional outputs of both the filamentous growth pathway and mating pathway. As yet the relevant substrates of Hog1 in cross-talk suppression have not been identified.

### Hog1 phosphorylates Ste50 to limit Fus3 activation

Ste50 is a shared component, required for activation of Ste11, that acts early in the mating and osmotic stress pathways. Thus Ste50 is well positioned to coordinate the activity of both Fus3 and Hog1. Moreover, Ste50 is phosphorylated by Hog1 and as a consequence of this phosphorylation there is an attenuated response to hyperosmotic stress [46], [47]. Here we show that as an additional consequence of Ste50 phosphorylation there is an attenuated response to pheromone. On that account, Ste50 is a target of both negative feedback during stress adaptation and cross-inhibition during co-stimulation.

The effects of co-stimulation are most evident at the level of the mating MAPKs. Salt-dependent phosphorylation of Ste50 attenuates pheromone-dependent activation of both Fus3 and Kss1. Whereas the phosphorylation of Ste50 fully accounts for cross-inhibition of Kss1, it is only partially responsible for cross-inhibition of Fus3. Consequently we searched for additional mechanisms of signal integration that act on Fus3 but not Kss1. Given that Fus3 is induced by pheromone - whereas Kss1 is not - we considered whether salt stress inhibits Fus3 transcription or translation.

### Hog1 phosphorylates Rck2 to limit Fus3 production

It was established previously that Hog1 directly phosphorylates and activates a repressor of translation elongation, Rck2 [42]. When Rck2 is absent, translation repression is abrogated [43]. Accordingly, we found that Rck2 is needed to inhibit Fus3 accumulation. As with Ste50^5A^, the effect of the *rck2*Δ mutation was incomplete. However combining both mutations (*rck2*Δ *ste50^5A^*) eliminated the ability of Hog1 to inhibit Fus3 (Figure S6). Thus Hog1 phosphorylates components necessary for the activation and induction of Fus3. Together these phosphorylation events act to limit mating responses as long as Hog1 is active. Once the cells are fully adapted, mating can proceed.

While it is clear that Rck2 confers a global inhibition of protein translation [43], it is important to note that Rck2 also contributes to the induction of distinct gene transcripts necessary for stress adaptation [51]. Thus Rck2 may represent a more general mechanism that ensures competing cellular processes do not interfere with the early translation of stress adaptive genes. It is also possible that Rck2 regulates other components of the pheromone pathway, in addition to Fus3. However, many of the core components that make up the MAPK cascade are stably expressed, including the scaffold (Ste5), the MAPKK (Ste7), the MAPKKK (Ste11) and its adaptor (Ste50) [9]. Therefore Hog1 and Rck2 are not expected to interfere with the ability to sense pheromone; rather Hog1 is likely to arrest signal transduction by those proteins that are induced by pheromone, most of which function downstream in the pathway (including Fus3). Thus we postulate that early components of the pheromone pathway are unaffected by hyperosmotic stress conditions.

### Ste5 sustains the mating response during stress adaptation

We propose that the earliest events in pheromone signaling, those not subject to pheromone mediated transcriptional induction, are unaffected by hyperosmotic stress conditions. These early events include G protein activation and recruitment of Ste5 [45]. Consistent with this view, our epistasis studies indicate that Hog1 acts downstream of the G protein. Moreover recruitment of Ste5 to the plasma membrane occurs even in the face of hyperosmotic stress [24]. This behavior suggests that the mating pathway remains quiescent only as long as conditions are unfavorable to launch a full mating response. Once cells adapt to stress, Hog1 is deactivated and mating can proceed immediately.

**Figure 7:**
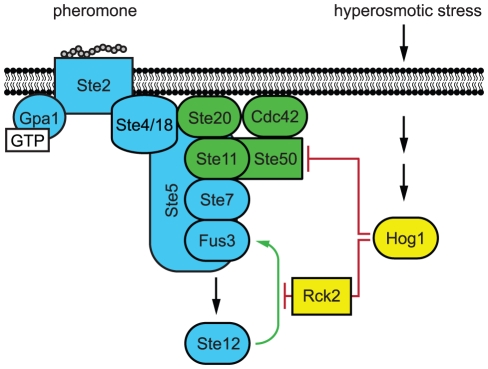
Model of Hog1 pathway cross-inhibition. Cells co-stimulated with mating pheromone and hyperosmotic stress adapt to stress before committing to mating differentiation. Hog1 coordinates mating and stress signals by limiting Fus3 activation through two mechanisms, (1) feedback phosphorylation of Ste50 and (2) feedforward phosphorylation of Rck2. Red lines indicate Hog1 mediated inhibition. Green line indicates the Fus3 positive feedback loop, which is disrupted by Rck2.

Taken together, available data support a model where mating and HOG pathways are both initiated in response to pheromones and hyperosmotic stress. However, the activation of Hog1 imparts a “checkpoint” midway in the pheromone signaling pathway, and does so to ensure quiescence of the mating response while cells adapt to stress (Figure 7). This design ensures that the mating pathway is primed to resume full signaling once Hog1 is no longer activated. Accordingly, Ste50 and Rck2 are both rapidly dephosphorylated upon adaptation [43], [46]. From these behaviors we can infer that scaffold proteins and shared adaptor proteins have distinct but complementary roles in signaling; scaffold proteins, epitomized by Ste5, behave as insulators, while shared components, such as Ste50, behave as dynamic integrators of multiple signals.

### Conclusions

As genomics and proteomics have defined signal pathway components, attention will turn increasingly to understanding how cells coordinate competing signals. In this regard, our findings reveal that pathway cross-inhibition is not a single process, but rather a network of events that work together to postpone cell differentiation until the cell adapts to stress conditions. More broadly, it is increasingly evident that a complete analysis of signal transduction networks will need to consider multiple inputs, multiple regulatory targets, and multiple mechanisms of action.

## Materials and Methods

### Strains, plasmids, and growth conditions

Standard procedures for growth, maintenance, and transformation of yeast and bacteria and for the manipulation of DNA were used throughout. Plasmids and strains were constructed as previously described [46], [52]–[55]. Yeast strains and plasmids used are listed in supplemental Table S7 and supplemental Table S8, respectively. All mutations were constructed with the QuikChange site-directed mutagenesis kit (Stratagene) according to the manufacturer's directions. Cells were grown in synthetic complete medium containing 2% (w/v) dextrose (SCD) or raffinose followed by the addition of 2% galactose to induce gene expression. Plasmid-transformed cells were grown in synthetic complete medium lacking the appropriate nutrient.

### Quantitative mating assay

Yeast mating efficiency was determined by a quantitative method, as described previously [56]. Cells were grown to OD_600_∼0.6 and counted using a hemocytometer. 5×10^6^ BY4741 (*MAT* **a** *his3*Δ1 *leu2*Δ0 *met15*Δ0 *ura3*Δ0) cells were mixed with 5×10^6^ BY4742 (*MAT*α *his3*Δ1 *leu2*Δ0 *lys2*Δ0 *ura3*Δ0) cells in a volume of 10 ml and passed over a nitrocellulose filter (Millipore). Filter disks were incubated for 4 h or 24 h on YPD agar or YPD agar containing 0.5 M KCl. Mating efficiency was calculated by dividing the number of diploid cells by the number of total cells after 4 h or 24 h mating period.

### Microscopy

Cells were grown to A_600 nm_∼0.8, dispersed by sonication with 10 pulses (1 sec, 50% output), and collected by centrifugation at 14,000× g for 15 seconds. 3 µl of cells were placed on glass slides coated with SCD medium 2% agar (w/v) and either α factor pheromone, or α factor and KCl. Cells were visualized every 15 min by differential interference contrast (DIC) and fluorescence microscopy using an Olympus Fluoview FV1000 confocal microscope with a 60× objective. GFP fluorescence was imaged using a 488-nm argon laser and 500–550 nm emission filter. Videos were constructed and images were analyzed using ImageJ (National Institutes of Health).

### Cell viability

Logarithmically growing cells (A_600 nm_∼0.6) were stimulated with 10 µM α factor and 0.75 M KCl or 1 M sorbitol. Viability was assessed by methylene blue staining (0.01% solution w/v) before and after 2 h of treatment. Prior to counting cells were dispersed by sonication with 5 pulses (1 sec, 50% output) and diluted 1∶10 in SCD with methylene blue and counted using a hemocytometer.

### Cell extracts and immunoblotting

Protein extracts were produced by glass bead lysis in TCA as previously described [55]. Protein concentration was determined by Dc protein assay (Bio-Rad Laboratories). Protein extracts were resolved by 7.5% or 12.5% SDS-PAGE and immunoblotting with HA antibodies (clone 3F10, Roche Applied Science) at 1∶2000, Phospho-p44/42 MAPK antibodies (9101, Cell Signaling Technology) at 1∶500, Fus3 antibodies (sc-6773, Santa Cruz Biotechnology, Inc.) at 1∶500, phospho-p38 MAPK antibodies (9216, Cell Signaling Technology) at 1∶500, Hog1 antibodies (sc-6815, Santa Cruz Biotechnology) at 1∶500, and glucose-6-phosphate dehydrogenase (G6PDH) antibodies (A9521, Sigma-Aldrich) at 1∶50,000. Far1-HA immunoreactive species were visualized by chemiluminescent detection (PerkinElmer Life Sciences LAS) of horseradish peroxidase-conjugated antibodies (sc-2006, Santa Cruz Biotechnology, Inc.) at 1∶10,000. All remaining immunoreactive species were visualized by fluorescent detection (Typhoon Trio+Imager, GE Healthcare) of AlexaFluor conjugated antibodies (A21245, A21424, A21431, Invitrogen) at 1∶2,000. Band intensity was quantified by scanning densitometry using Image J (National Institutes of Health). P-Fus3 and P-Kss1 values were normalized to G6PDH loading control.

### Transcriptional reporter assay

*FUS1-LacZ* levels were measured every 30 min after treatment with mating pheromone α factor, or α factor and KCl or sorbitol using a β-galactosidase assay as described previously [54]. Cells were split and diluted 30% with fresh medium containing pheromone alone or pheromone and an indicated concentration of KCl or sorbitol. Aliquots of cells were removed every 30 min, lysed, and β-galactosidase activity was measured.

## Supporting Information

Figure S1 Sorbitol dampens and delays the mating response. Transcriptional activation (β-galactosidase activity) was measured spectrofluorometrically every 30 min in cells transformed with plasmid containing a pheromone-inducible reporter (*FUS1*-lacZ). Transcription was induced by the addition of 10 µM α factor, 10 µM α factor+0.75 M sorbitol, and 10 µM α factor+1.5 M sorbitol. Data are the mean ± SE of four individual colonies measured in quadruplicate and presented as percentage of wild-type maximum.
(EPS)

Figure S2 High osmolarity has minor effects on pheromone sensitivity. Transcriptional activation (β-galactosidase activity) was measured spectrofluorometrically at (A) 90 and (B) 180 min in cells transformed with plasmid containing a pheromone-inducible reporter (*FUS1*-lacZ). Transcription was induced by the addition of α factor with 0.75 M KCl or 0.75 M sorbitol. Data are the mean ± SE of four individual colonies measured in quadruplicate and presented as percentage of wild-type maximum.
(EPS)

Figure S3 Hog1 activation is required to disrupt the pheromone response. (A) Cells treated with sub-threshold concentrations of KCl (0.005 and 0.05 M) and 0.5 M KCl together with 10 µM α factor for 60 min. Cell lysates were resolved by 12.5% SDS-PAGE. P-Fus3 and P-Kss1 were detected with phospho-p44/p42 antibodies. P-Hog1 was detected with phospho-p38 antibodies. Total Fus3 and Hog1 were detected with Fus3 and Hog1 antibodies. G6PDH served as a loading control. All primary antibodies were recognized by fluorescently labeled secondary antibody, detected by fluorescence scanner (Typhoon Trio) and quantified by scanning densitometry (ImageJ). The panel to the right shows averaged scanning densitometry of three individual experiments. Error bars represent ± SEM. (B) Transcriptional activation (β-galactosidase activity) was measured spectrofluorometrically at 120 min in cells transformed with plasmid containing a pheromone-inducible reporter (*FUS1*-lacZ). Transcription was induced by the addition of 30 µM α factor and varying concentrations of KCl. Data are the mean ± SE of three individual experiments and presented as percentage of cells treated with 30 µM α factor alone.
(EPS)

Figure S4 Constitutively active Hog1 diminishes stably expressed Fus3. *fus3*Δ cells transformed with *GAL1*-*FUS3* and *GAL1*-*SSK2^ΔN^* or vector were grown and stimulated as in Figure 5C. P-Hog1 reduced P-Fus3 by 25.4%±12.8%. Increased experimental variability may be attributed to promoter competition.
(EPS)

Figure S5 Ste50 is required for full activation of the pheromone pathway. Wild-type or *ste50*Δ cells transformed with either *GAL1*-*STE5^CTM^* or *GAL1*-*STE11^ΔN^* were grown in 2% raffinose for 90 min. Cell lysates were resolved by 12.5% SDS-PAGE. P-Fus3 and P-Kss1 were detected with phospho-p44/p42 antibodies. Fus3 was detected with Fus3 antibodies. G6PDH served as a loading control. All primary antibodies were recognized by fluorescently labeled secondary antibody and quantified. The panels to the right show averaged scanning densitometry of three individual experiments. Error bars represent ± SEM.
(EPS)

Figure S6 Rck2 and Ste50 limit mating MAPK activation and induction. Graphical representation of western blot quantification from Figure 5B and 5C, Figure 6E and 6F. Data are plotted relative to maximum activation of pheromone treated cells.
(EPS)

Figure S7 The effect of Hog1 on Fus3 during stress adaptation is diminished in the *ste50^5A^ rck2*Δ mutant. Sequential stimulation of Hog1 and Fus3 in *ste50^5A^ rck2*Δ cells grown in SC with 2% dextrose stimulated with 0.75 M KCl, and after an indicated period of stress adaptation stimulated with 10 µM α factor for an additional 15 min. Error bars represent ± SEM. Overlaid red columns represent wild-type response during the same conditions as presented in Figure 4C.
(EPS)

Table S1 Wild-type α factor response time course; see Figure 2A and Figure S1.
(DOC)

Table S2 hog1Δ α factor response time course; see Figure 2B.
(DOC)

Table S3 Hog1^K52R^ α factor response time course; see Figure 2C.
(DOC)

Table S4 Wild-type α factor dose response log EC_50_, M; see Figure S2.
(DOC)

Table S5 Wild-type α factor response time course (*FUS1*-GFP); see Figure 2E.
(DOC)

Table S6 Hyperosmotic stress decreases mating efficiency.
(DOC)

Table S7 Strains used in this study.
(DOC)

Table S8 Plasmids used in this study.
(DOC)

Text S1 Analysis of additional effects associated with hyperosmotic stress on the mating response.
(DOC)

Video S1 Shmoo formation with 100 µM α factor. The video accompanies Figure 1B and represents images captured over 300 min, every 15 min.
(AVI)

Video S2 Shmoo formation with 100 µM α factor+0.5 M KCl. The video accompanies Figure 1B and represents images captured over 300 min, every 15 min.
(AVI)

Video S3 Shmoo formation with 100 µM α factor+0.75 M KCl. The video accompanies Figure 1B and represents images captured over 300 min, every 15 min.
(AVI)
