# Supplementary material for: Checkpoints in a Yeast Differentiation Pathway Coordinate Signaling during Hyperosmotic Stress
Source: PLoS Genet. 2012 Jan 5;8(1):e1002437. doi: 10.1371/journal.pgen.1002437 (PMC3252264; doi:10.1371/journal.pgen.1002437)
Supplement: Table S1 — Wild-type α factor response time course; see Figure 2A and Figure S1. (DOC) [file pgen.1002437.s008.doc]

Table S1. Wildtype α factor response time course; see Figure 2A and Figure S1

| stimulus | t _½ max_ (min) | basal  response* | maximum response* |
| --- | --- | --- | --- |
| 10 μM α factor | 49.3 ± 3.3 | 1.3% ± 0% | 100.3% ± 5.4% |
| 10 μM α factor +  0.5 M KCl | 61.7 ± 2.2 | 1.3% ± 0% | 88.9% ± 3.7% |
| 10 μM α factor +  0.75 M KCl | 82.5 ± 0.8 | 1.3% ± 0.1% | 82.9% ± 6.7% |
| 10 μM α factor +  1 M KCl | 100.7 ± 0.8 | 1.3% ± 0% | 67.8% ± 5.6% |
| 10 μM α factor +  0.75 M sorbitol | 67.0 ± 6.6 | 1.3% ± 0.1% | 94% ± 7.3% |
| 10 μM α factor +  1.5 M sorbitol | 156.7 ± 11.1 | 1.4% ± 0.1% | 68.6% ± 0.3% |

* percent of maximum response
