# Supplementary material for: Checkpoints in a Yeast Differentiation Pathway Coordinate Signaling during Hyperosmotic Stress
Source: PLoS Genet. 2012 Jan 5;8(1):e1002437. doi: 10.1371/journal.pgen.1002437 (PMC3252264; doi:10.1371/journal.pgen.1002437)
Supplement: Table S2 — hog1Δ α factor response time course; see Figure 2B. (DOC) [file pgen.1002437.s009.doc]

Table S2. hog1Δ α factor response time course; see Figure 2B

| stimulus | t _½ max_ (min) | basal  response* | maximum response* |
| --- | --- | --- | --- |
| 10 μM α factor | 42.4 ± 1.6 | 1.3% ± 0.1% | 92.3% ± 3.4% |
| 10 μM α factor +  0.5 M KCl | 34.0 ± 2.8 | 1.3% ± 0.1% | 100.9% ± 2.6% |
| 10 μM α factor +  0.75 M KCl | 54.0 ± 2.0 | 1.3% ± 0.1% | 101.2% ± 1.8% |
| 10 μM α factor +  1 M KCl | 89.7 ± 1.3 | 1.3% ± 0.1% | 97.6% ± 3.4% |

* percent of wildtype maximum response from Table S1
