# Supplementary material for: Checkpoints in a Yeast Differentiation Pathway Coordinate Signaling during Hyperosmotic Stress
Source: PLoS Genet. 2012 Jan 5;8(1):e1002437. doi: 10.1371/journal.pgen.1002437 (PMC3252264; doi:10.1371/journal.pgen.1002437)
Supplement: Table S3 — Hog1K52R α factor response time course; see Figure 2C. (DOC) [file pgen.1002437.s010.doc]

Table S3. Hog1^K52R^ α factor response time course; see Figure 2C

| stimulus | t _½ max_ (min) | basal  response* | maximum response* |
| --- | --- | --- | --- |
| 10 μM α factor | 48.9 ± 2.5 | 4.1% ± 0.9% | 113.6% ± 2.9% |
| 10 μM α factor +  0.5 M KCl | 41.6 ± 2.9 | 4.1% ± 0.9% | 118.5% ± 3.8% |
| 10 μM α factor +  0.75 M KCl | 71.7 ± 1.1 | 4.1% ± 0.9% | 119.7% ± 3.7% |
| 10 μM α factor +  1 M KCl | 104.8 ± 1.0 | 4.1% ± 0.9% | 114.2% ± 5.4% |

* percent of wildtype maximum response from Table S1
