# Supplementary material for: Checkpoints in a Yeast Differentiation Pathway Coordinate Signaling during Hyperosmotic Stress
Source: PLoS Genet. 2012 Jan 5;8(1):e1002437. doi: 10.1371/journal.pgen.1002437 (PMC3252264; doi:10.1371/journal.pgen.1002437)
Supplement: Table S4 — Wild-type α factor dose response log EC50, M; see Figure S2. (DOC) [file pgen.1002437.s011.doc]

Table S4. Wildtype α factor dose response log EC_50_, M; see Figure S2

| time treated | no osmolyte | 0.75 M KCl | 0.75 M sorbitol |
| --- | --- | --- | --- |
| 90 minutes | -5.74 ± 0.08 | -5.92 ± 0.11 | -6.25 ± 0.15 |
| 180 minutes | -5.62 ± 0.07 | -5.30 ± 0.06 | -5.68 ± 0.12 |
