# Supplementary material for: Checkpoints in a Yeast Differentiation Pathway Coordinate Signaling during Hyperosmotic Stress
Source: PLoS Genet. 2012 Jan 5;8(1):e1002437. doi: 10.1371/journal.pgen.1002437 (PMC3252264; doi:10.1371/journal.pgen.1002437)
Supplement: Table S5 — Wild-type α factor response time course (FUS1-GFP); see Figure 2E. (DOC) [file pgen.1002437.s012.doc]

Table S5. Wildtype α factor response time course (*FUS1*-GFP); see Figure 2E

| stimulus | t _½ max_ (min) | maximum response |
| --- | --- | --- |
| 100 μM α factor | 100.8 ± 2.1 | 96.5% ± 0.5% |
| 100 μM α factor +  0.5 M KCl | 118.9 ± 2.8 | 84.8% ± 0.8% |
