# Supplementary material for: Checkpoints in a Yeast Differentiation Pathway Coordinate Signaling during Hyperosmotic Stress
Source: PLoS Genet. 2012 Jan 5;8(1):e1002437. doi: 10.1371/journal.pgen.1002437 (PMC3252264; doi:10.1371/journal.pgen.1002437)
Supplement: Table S6 — Hyperosmotic stress decreases mating efficiency. (DOC) [file pgen.1002437.s013.doc]

Table S6. Hyperosmotic stress decreases mating efficiency

|  | mating efficiency (%)* | |
| --- | --- | --- |
| **a** strain | YPD | YPD + 0.5 M KCl |
| wildtype | 77 ± 14 | 28 ± 5 |
| ste50^5A^ | 88 ± 18 | 47 ± 9 |
| rck2Δ | 84 ± 15 | 34 ± 6 |
| ste50^5A^ rck2Δ | 80 ± 13 | 49 ± 9 |

* mating efficiency was calculated by dividing the number of diploid cells by the number of total cells after 4 hour mating. BY4741 **a** strains were mated with a wildtype α strain, BY4742
