# Supplementary material for: Checkpoints in a Yeast Differentiation Pathway Coordinate Signaling during Hyperosmotic Stress
Source: PLoS Genet. 2012 Jan 5;8(1):e1002437. doi: 10.1371/journal.pgen.1002437 (PMC3252264; doi:10.1371/journal.pgen.1002437)
Supplement: Table S7 — Strains used in this study. (DOC) [file pgen.1002437.s014.doc]

Table S7. Strains used in this study

| Strain Name | Genotype | Source |
| --- | --- | --- |
| BY4741* | *MAT***a** *leu2*Δ *met15*Δ *his3-1 ura3*Δ | [52] |
| BY4742 | *MAT*α *leu2*Δ *lys2*Δ *his3-1 ura3*Δ | [52] |
| *hog1*Δ | *MAT***a*** hog1::*kanMX | Invitrogen |
| *fus3*Δ | *MAT***a*** fus3::*kanMX | Invitrogen |
| *rck2*Δ | *MAT***a*** rck2::*kanMX | Invitrogen |
| *hog1^K52R^* | *MAT***a*** hog1^K52R^* | [56] |
| *ste50^5A^* | *MAT***a*** ste50^5A^* | This study, see [46] |
| *ste50^5A^* *rck2*Δ | *MAT***a*** ste50^5A^ rck2::*kanMX | This study |

* All mutant strains derived from BY4741
