# Supplementary material for: Checkpoints in a Yeast Differentiation Pathway Coordinate Signaling during Hyperosmotic Stress
Source: PLoS Genet. 2012 Jan 5;8(1):e1002437. doi: 10.1371/journal.pgen.1002437 (PMC3252264; doi:10.1371/journal.pgen.1002437)
Supplement: Table S8 — Plasmids used in this study. (DOC) [file pgen.1002437.s015.doc]

Table S8. Plasmids used in this study

| Fig | Plasmid Name | Description | Source |
| --- | --- | --- | --- |
| 6 | pRS413 GAL1/10 | CEN HIS3 P_GAL1/10_ vector | Henrik Dohlman |
| 5,6 | pYES2.1 | 2µ URA3 vector | Invitrogen |
| 1 | pRS316 FAR1-HA | CEN URA3 FAR1-HA | Jenna Slessareva |
| 2 | pRS423 FUS1-lacZ | 2µ HIS3 P_FUS1_-lacZ | [54] |
| 4 | pYES2.1 GAL1-FUS3 | 2µ URA3 P_GAL1_-FUS3 | This study |
| 5 | pRS315 ADH1-FUS3 | 2µ LEU2 P_ADH1_-FUS3 | This study |
| 5 | pYES2.1 GAL1-SSK2-ΔN | 2µ URA3 P_GAL1_-SSK2-ΔN | This study |
| 6 | pRS413 GAL1-SSK2-ΔN | CEN HIS3 P_GAL1_-SSK2-ΔN | This study |
| 6 | pRS313 GAL1-STE5^CTM^ | CEN HIS3 P_GAL1_-STE5^CTM^ | [53] |
| 6 | pYES2.1 GAL1-STE11-ΔN | 2µ URA3 P_GAL1_-STE11-ΔN | Beverly Errede |
