# Supplementary material for: Checkpoints in a Yeast Differentiation Pathway Coordinate Signaling during Hyperosmotic Stress
Source: PLoS Genet. 2012 Jan 5;8(1):e1002437. doi: 10.1371/journal.pgen.1002437 (PMC3252264; doi:10.1371/journal.pgen.1002437)
Supplement: Text S1 — Analysis of additional effects associated with hyperosmotic stress on the mating response. (DOC) [file pgen.1002437.s016.doc]

**Supporting Text**

**Hyperosmotic conditions do not disrupt pheromone-receptor interactions**

High concentrations of any osmolyte could disrupt protein-protein interactions raising the concern that salt conditions might disrupt binding of a peptide pheromone to its receptor. Binding can be assessed by determining the effective pheromone concentrations that give half-maximum response (EC50). We determined the EC50 of pheromone with the addition of KCl or sorbitol by measuring *FUS1*-lacZ activity (Figure S2 and Table S4). To distinguish between cells undergoing stress-adaptation from those that are fully adapted, we determined the EC50 near the t_½ max_ (90 min) and also after 180 minutes (Figure 2A). In this comparison we found no significant differences in EC50. Thus hyperosmotic stress does not diminish sensitivity to pheromone.

**The mating response is insensitive to low concentrations of osmolyte**

Hog1 activation by salt stress is ultrasensitive [1,2]. Accordingly, sub-threshold concentrations of KCl (< 0.2 M) do not activate Hog1. Our model predicts that active Hog1 limits the mating response, thus we aimed to determine if low salt concentrations also diminish pheromone signaling. As shown in Figure S3 we measured both Fus3 phosphorylation and *FUS1*-lacZ activity in the presence of non-activating and Hog1-activating concentrations of KCl. Addition of 0.005 and 0.05 M KCl did not activate Hog1 or diminish Fus3, while 0.5 M KCl activated Hog1 and diminished Fus3 (Figure S3A). Similarly *FUS1-GFP* activity was not dampened by low sorbitol concentration [3], and *FUS1*-lacZ activity was not dampened by low KCl concentrations whereas higher concentrations reduced pathway output (Figure S3B). Thus we conclude that salt alone is not sufficient to limit the mating response, while Hog1 activation in response to hyperosmotic stress limits mating.

**Supporting References**

1. Brewster JL, de Valoir T, Dwyer ND, Winter E, Gustin MC (1993) An osmosensing signal transduction pathway in yeast. Science 259: 1760-1763.

2. Maeda T, Takekawa M, Saito H (1995) Activation of yeast PBS2 MAPKK by MAPKKKs or by binding of an SH3-containing osmosensor. Science 269: 554-558.

3. Patterson JC, Klimenko ES, Thorner J (2010) Single-cell analysis reveals that insulation maintains signaling specificity between two yeast MAPK pathways with common components. Sci Signal 3: ra75.
